# Supplementary material for: A specific microbial consortium enhances Th1 immunity, improves LCMV viral clearance but aggravates LCMV disease pathology in mice
Source: Nat Commun. 2025 Apr 25;16:3902. doi: 10.1038/s41467-025-59073-x (PMC12022176; doi:10.1038/s41467-025-59073-x)
Supplement: Supplementary file 1 — Supplementary Information [file 41467_2025_59073_MOESM1_ESM.pdf]

Supplementary Figure 1-8

for

A specific microbial consortium enhances Th1 immunity, improves LCMV viral clearance but aggravates LCMV disease pathology in mice

Running title: Microbial impact on Th1 immunity

Daphne Kolland[1], Miriam Kuhlmann[2], Gustavo P. de Almeida[2], Amelie Köhler[1], Anela Arifovic[1], Alexandra von Stempel[3], Mohsen Pourjam[4], Silvia Bolsega[5], Christine Wurmser[2], Katja Steiger[6], Marijana Basic[5], Klaus Neuhaus[4], Carsten B. Schmidt-Weber[1], Bärbel Stecher[3], Dietmar Zehn[2] *, Caspar Ohnmacht[1]*

[1] Center of Allergy and Environment (ZAUM), Technical University and Helmholtz Center Munich, Germany.
[2] Division of Animal Physiology and Immunology, School of Life Sciences Weihenstephan, Technical University of Munich, Freising, Germany.
Center for Infection Prevention (ZIP), School of Life Sciences Weihenstephan, Technical University of Munich, Freising, Germany
[3] Max von Pettenkofer Institute of Hygiene and Medical Microbiology, Faculty of Medicine, LMU Munich, Germany.
[4] Core Facility Microbiome ZIEL – Institute for Food & Health, Technical University of Munich, Freising, Germany.
[5] Institute for Laboratory Animal Science and Central Animal Facility, Hannover Medical School, 30625, Hannover, Germany.
[6] Institute of Pathology, School of Medicine and Health, Technical University Munich, Munich, Germany.
[7] Member of the German Center of Lung Research (DZL), Partner Site Munich, Munich, Germany.
[8] German Center for Infection Research (DZIF), partner site LMU Munich, Germany.*

***Corresponding authors:**
Dietmar Zehn, Division of Animal Physiology and Immunology, School of Life Sciences Weihenstephan, Technical University of Munich, Freising, Germany.

Email: dietmar.zehn@tum.de

Caspar Ohnmacht, Center of Allergy and Environment (ZAUM), Technical University and Helmholtz Center Munich, Germany.

Email: caspar.ohnmacht@helmholtz-munich.de

Supplementary Figures

Supplementary Figure 1

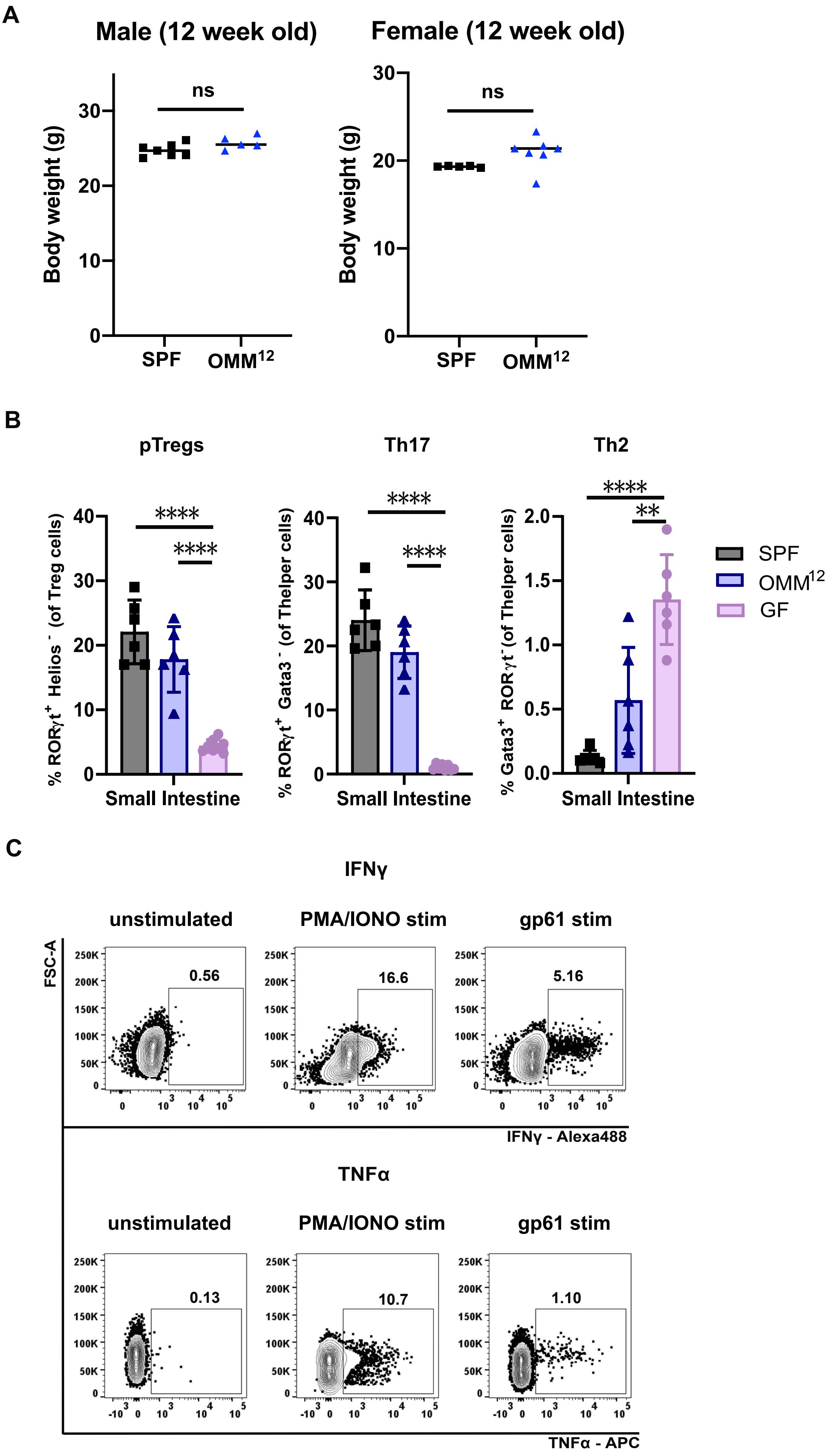

Supplementary Figure 1: Microbial impact on body weight and intestinal CD4+ T cell subsets.

A) Body weight of SPF (black quadrants) and OMM12 (blue triangles) male (left) and female (right) mice at 12 weeks of age. SPF n = 5-7, OMM12 n = 5-7. Each dot represents an individual mouse and mean ± SD is shown. Statistical analysis was performed using two-tailed students t-test. *P* value of <0.05 was considered statistically significant with *$p < 0.05$, **$p < 0.01$, ***$p < 0.001$, ****$p < 0.0001$. Source data are provided as a Source Data file. B and C) SPF (grey), OligoMM12-colonized (OMM12, blue) and germfree (GF, pink) mice were intravenously infected with LCMV Clone-13 and analyzed day 8 post infection (dpi). **B**) Cell frequencies of pTregs, Th17 and Th2 cells in small intestine lamina propria. *P* values are as followed, for pTregs in small intestine (SPF vs GF < 0.0001, OMM12 vs GF < 0.0001), for Th17 in small intestine (SPF vs GF < 0.0001 and OMM12 vs GF < 0.0001), for Th2 in small intestine (SPF vs GF < 0.0001 and OMM12 vs GF = 0.0041). Each dot represents an individual mouse and mean ± SD from two independent experiments is shown (n = 6). Statistical analysis was performed using two-tailed students t-test. *P* value of <0.05 was considered statistically significant with *$p < 0.05$, **$p < 0.01$, ***$p < 0.001$, ****$p < 0.0001$. Source data are provided as a Source Data file. **C**) Representative flow cytometry plots of unstimulated (left), PMA/IONO restimulated (middle) or virus-peptide gp61 (right) restimulated $CD4^{+}$ T cells. Each dot represents an individual mouse and mean ± SD from two independent experiments is shown. Source data are provided as a Source Data file.

Supplementary Figure 2

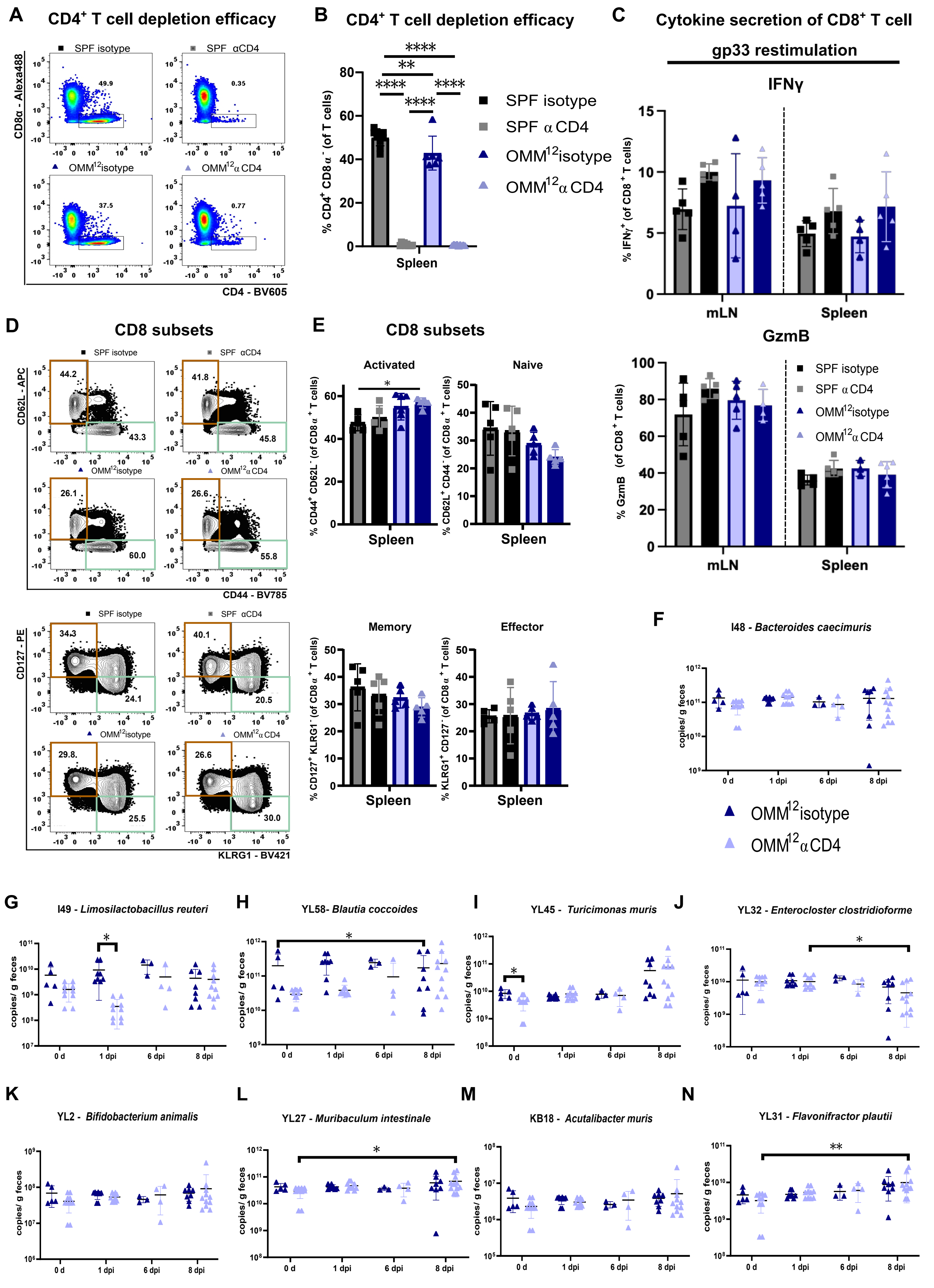

Supplementary Figure 2. Effect of CD4$^+$ T cell depletion on CD8 T cells and OMM^{12} microbial stability during LCMV Cl-13 infection.

SPF (grey) and OligoMM12-colonized (OMM^{12}, blue) mice were treated one day before, on the day of the clone-13 LCMV infection and 6 days post infection with either a depleting anti-CD4 or an isotype antibody control as illustrated in **Fig. 2A**. **A**) Representative flow cytometry plots and **B**) cell frequencies of splenic CD4+ T cells on day 8 post infection (*n*=6). P values are as followed, in the spleen(SPF isotype vs SPFantiCD4 < 0.00001, SPF antiCD4 vs OMM^{12} isotype < 0.00001, OMM^{12} isotype vs OMM^{12} antiCD4 < 0.00001, SPF isotype vs OMM^{12} antiCD4 < 0.00001 and SPF isotype vs OMM^{12} isotype = 0.00141). **C**) Cell frequencies of cytokine+ total CD8+ T cells spleen and mLN after re-stimulation with gp33. Representative flow cytometry plots (**D**) and cell frequencies (**E**) of CD8+ T cell subsets in spleen (*n*=5). *P* values are as followed activated CD8 subsets in spleen (SPF isotype vs OMM^{12} antiCD4 = 0.0339) **F-N**) Colonization dynamics of OMM^{12} mice during infection and after CD4 depletion: absolute abundance per gram feces of individual OMM^{12} strains at the indicated time points are shown. Each dot represents an individual mouse and mean ± SD from two independent experiments is shown. *P* value is as followed **G**) on 1 dpi (OMM12 isotype vs OMM12 antiCD4 = 0.0041) **H**) in OMM^{12} isotype (0d vs 8 dpi = 0.0047), **I**) at 0d (OMM^{12} isotype vs OMM^{12} antiCD4 = 0.0125), **J**) in OMM^{12} antiCD4 (1 dpi vs 8 dpi = 0.0348), **L**) in OMM^{12} antiCD4 (0d vs 8 dpi = 0.0282) and **N**) in OMM^{12} antiCD4 (0d vs 8 dpi = 0.0073) . Data from SPF (ctr) n = 5, SPF (anti-CD4) n =5, OMM^{12} (ctr) n = 6, OMM^{12} (anti-CD4) n = 5 mice are shown (**A-E**). Statistical analysis was performed using two-tailed students t-test. *P* value of <0.05 was considered statistically significant with *p < 0.05, **p < 0.01, ***p < 0.001, ****p < 0.0001. Source data are provided as a Source Data file.

Supplementary Figure 3

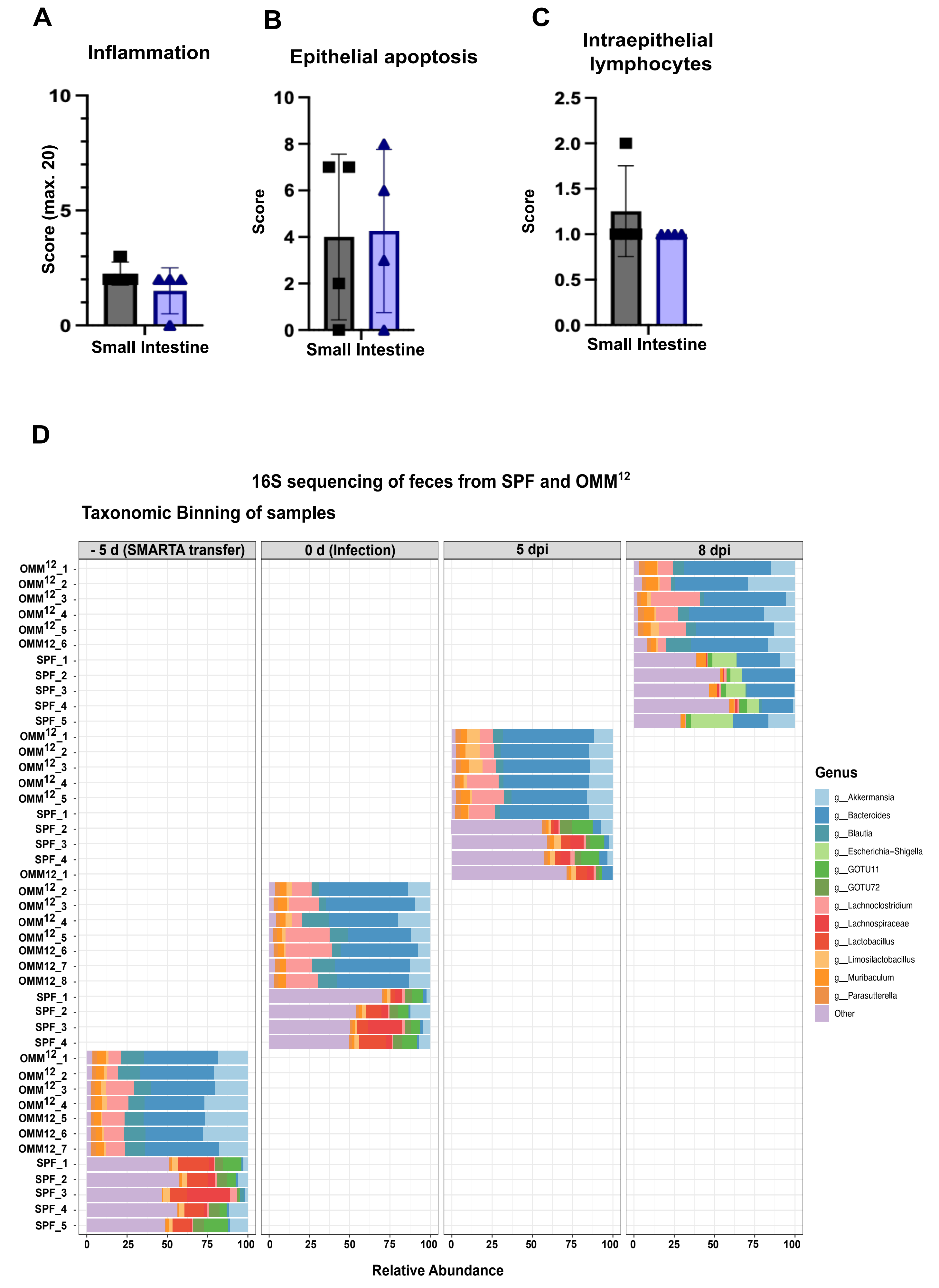

Supplementary Figure 3. No intestinal inflammation in OMM12 animals during LCMV infection and relative stability of OMM12 bacterial community.

Bar plots depict histological examination of intestinal inflammation of SPF and OMM12-colonized mice after LCMV Cl-13 infection showing **A**) inflammation score, **B**) epithelial apoptosis and **C**) intraepithelial lymphocyte infiltration. Feces from n = 3-10 OligoMM12-colonized and SPF mice was collected on the day of adoptive SMARTA T cell transfer (-5 d), on the day of infection with LCMV-Cl-13 (0 d), 5 days post infection (5 dpi) and on the day of analysis (8 dpi). **D**) Graph depicts relative genus-level distribution of assigned operational taxonomic units (OTUs) for isolated gDNA from feces made with Namco Microbiome Explorer (v1.1). Data from SPF n = 4, OMM12 n = 4 mice are shown (**A-C**). Data from SPF n = 5, OMM12 n = 7 mice are shown (**D**).

Supplementary Figure 4

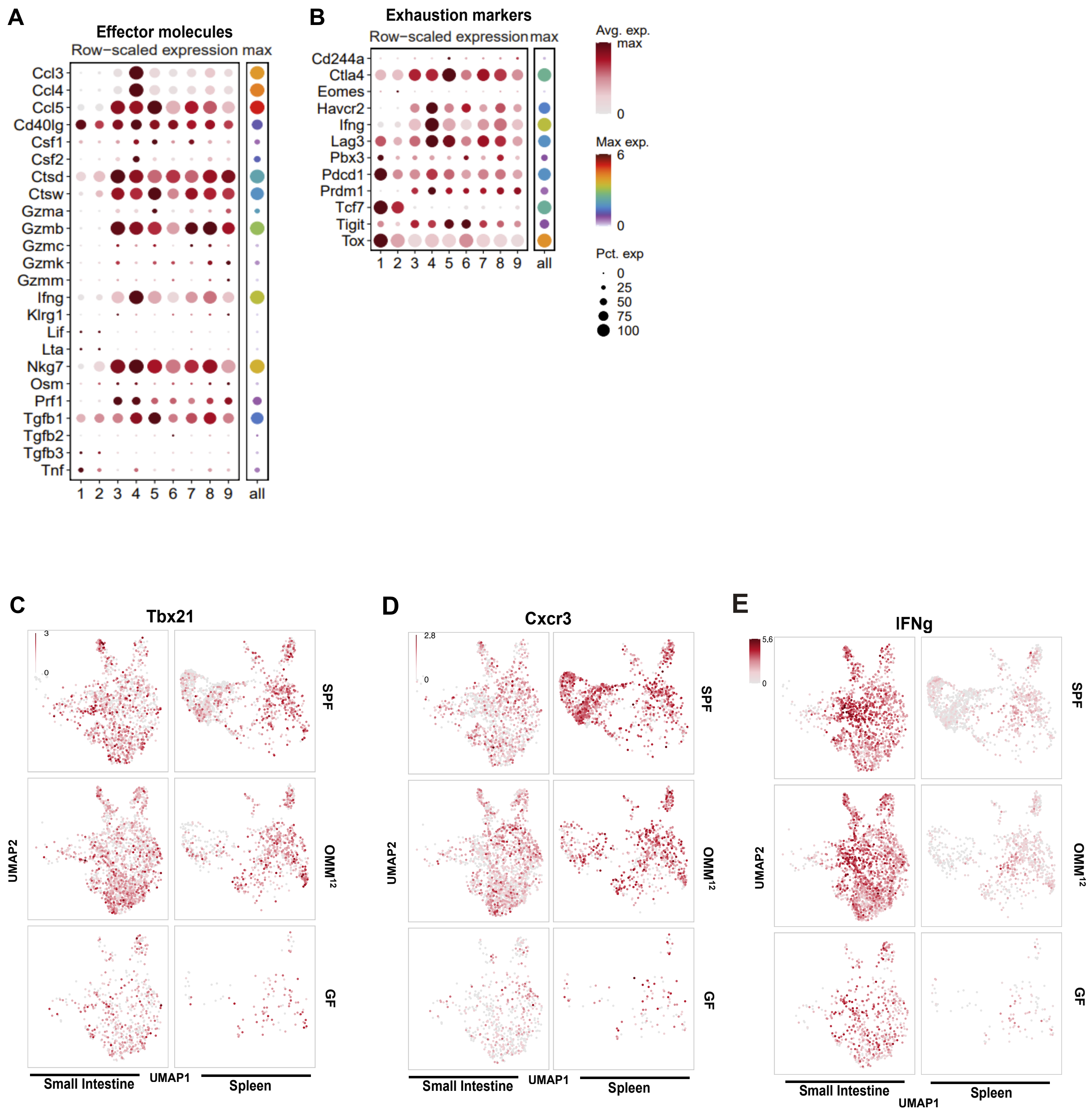

Supplementary Figure 4. Expression of effector molecules and T cell exhaustion markers in individual clusters and individual gene expression across organs and microbial status.
Feature plot of the average expression and percentage of cells from each cluster for effector molecules (**A**) T cell exhaustion markers (**B**) of re-isolated SMARTA T cells after single-cell RNAseq analysis. Expression of *Tbx21* (**C**) *Cxcr3* (**D**) and *Ifng* (**E**) on each individual cell for each sample group represented over the reduced space of UMAP. Refers to **Figure 4** and **5**.

Supplementary Figure 5

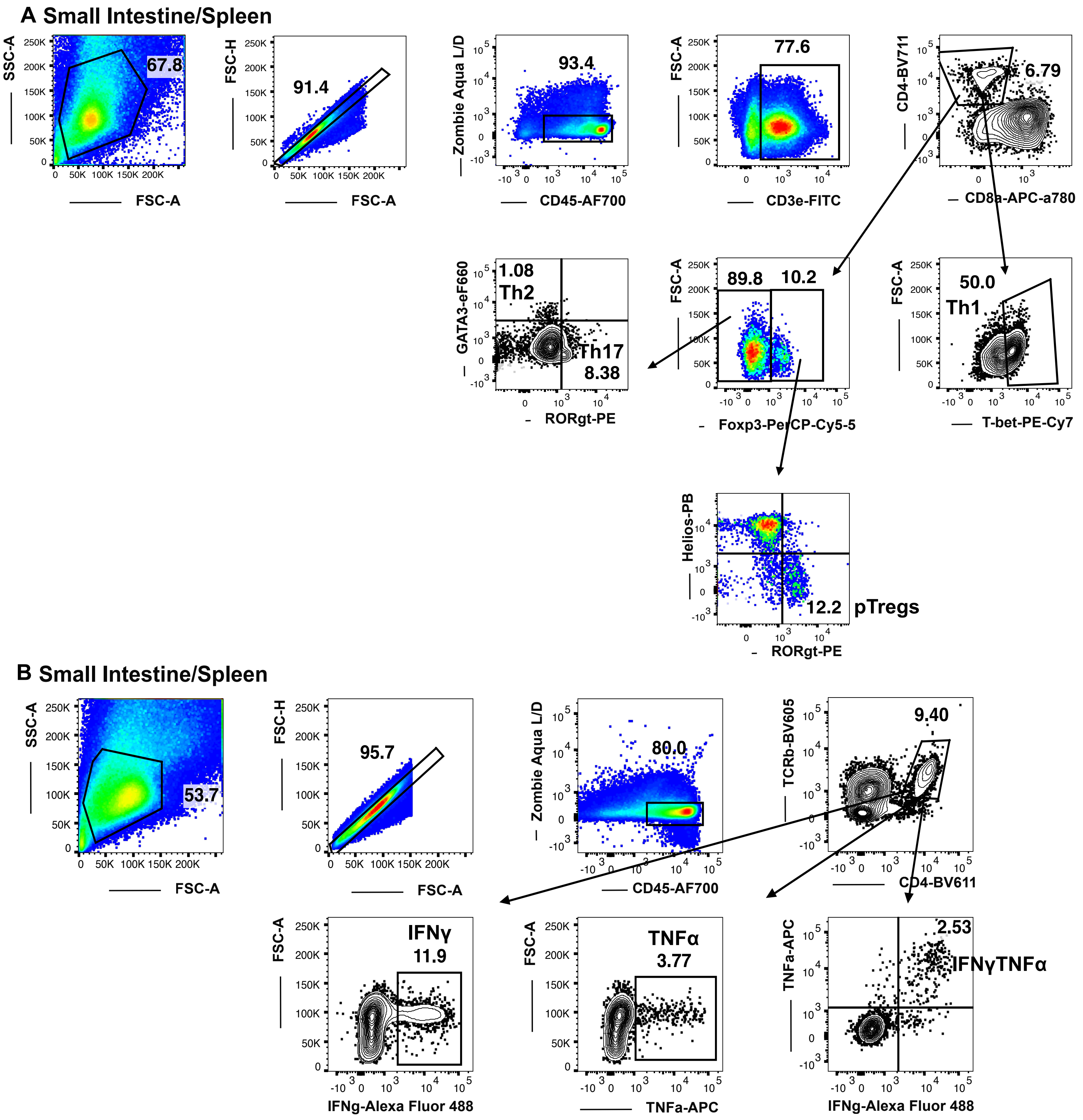

Supplementary Figure 5. Gating strategy for the identification of endogenous Th1 cells and cytokine secretion of gp61 stimulated CD4$^+$ T cells

A) Representative flow cytometry plots showing gating strategy for the identification of T-bet+ Th1 cells in small intestine lamina propria. effector molecules **B**) Representative flow cytometry plots showing gating strategy for the identification of cytokine$^+$ (IFNγ$^+$TNFα$^+$, IFNγ$^+$, TNFα$^+$) gp61 stimulated CD4+ T cells in small intestine lamina propria.

Supplementary Figure 6

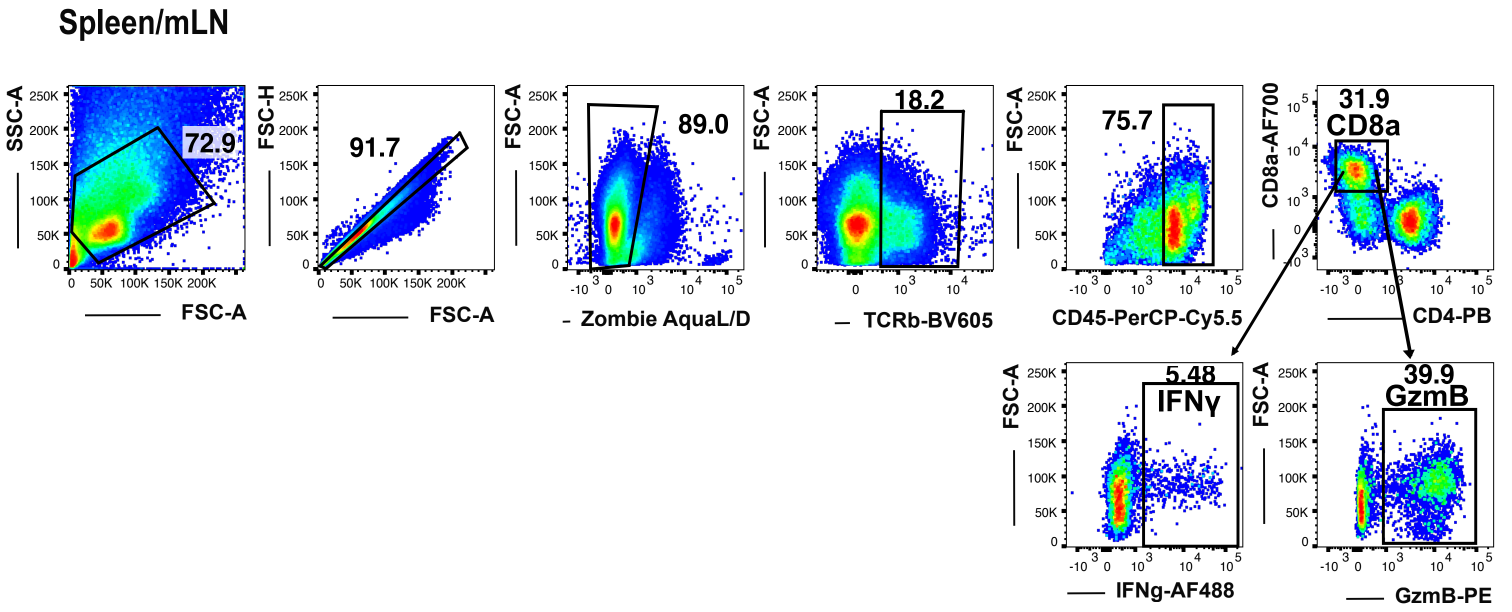

Supplementary Figure 6. Gating strategy for the identification of CD4⁺ T cells and cytokine secreting CD8a⁺ T cells

Representative flow cytometry plots showing gating strategy for the identification of CD4⁺ T cells cells and of cytokine⁺(IFNγ⁺,GzmB⁺) gp33 stimulated CD8a⁺ T cells in the spleen.

Supplementary Figure 7

A Small intestine/Spleen

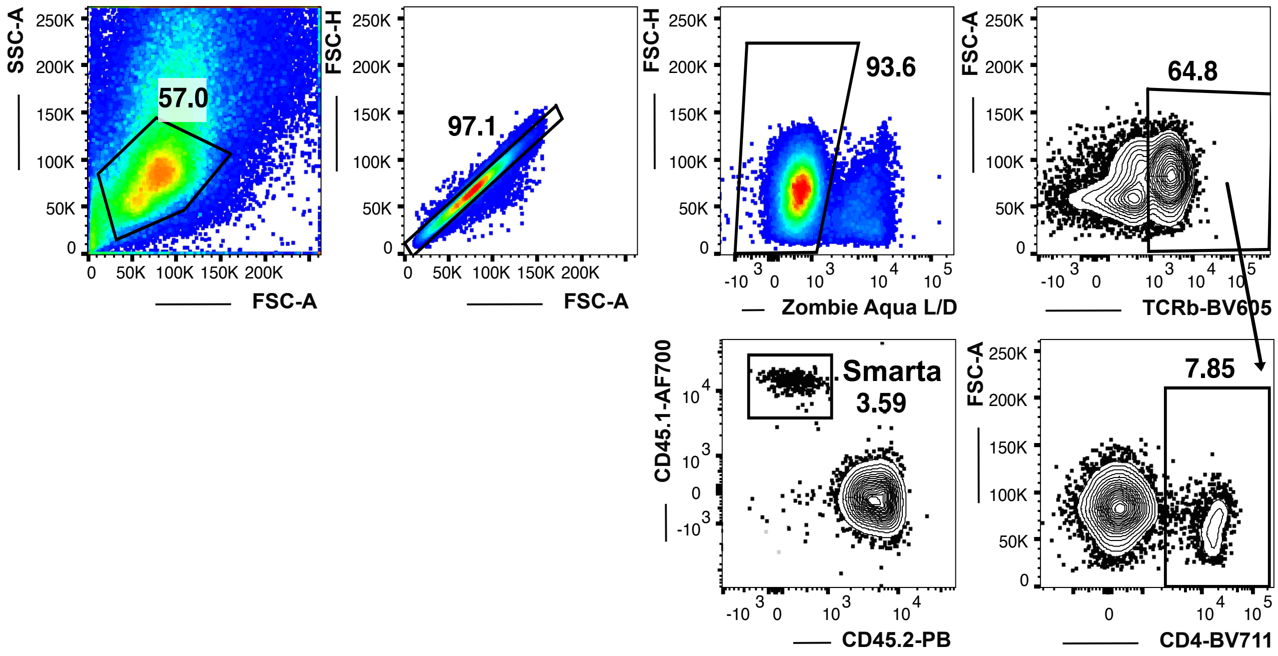

B Small Intestine/Spleen

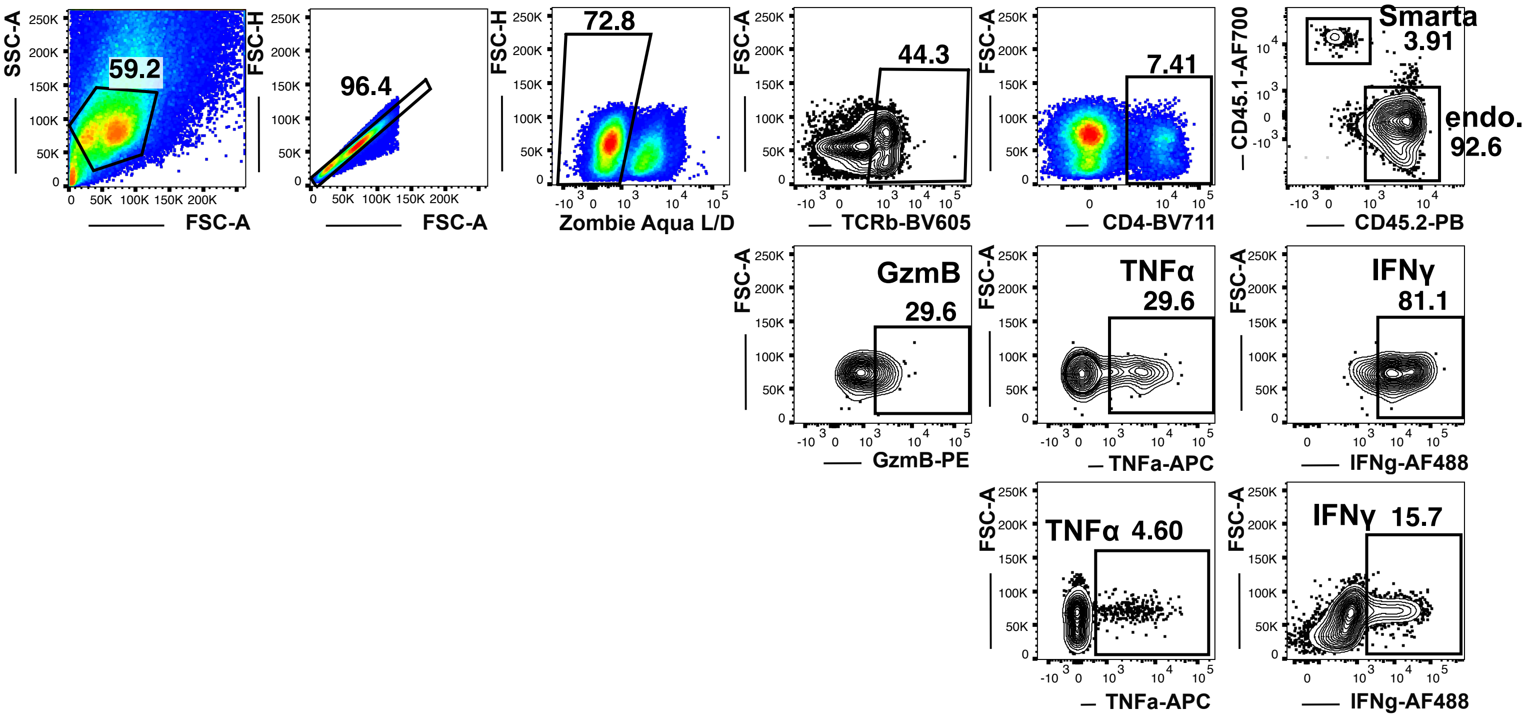

Supplementary Figure 7. Gating strategy for the identification of adoptively transferrred Smarta cells and cytokine secretion of gp61 stimulated Smarta cells and endogenous CD4⁺ T cells.

A) Representative flow cytometry plots showing gating strategy for the identification of adoptively transferred Smarta cells in small intestine lamina propria. **B**) Representative flow cytometry plots showing gating strategy for the identification of cytokine$^+$ (IFNγ$^+$, TNFa$^+$, GzmB$^+$) gp61 stimulated adoptively transferred Smarta cells and cytokine$^+$ (IFNγ$^+$, TNFa$^+$) of endogenous CD4$^+$ T cells in the small intestine.

Supplementary Figure 8

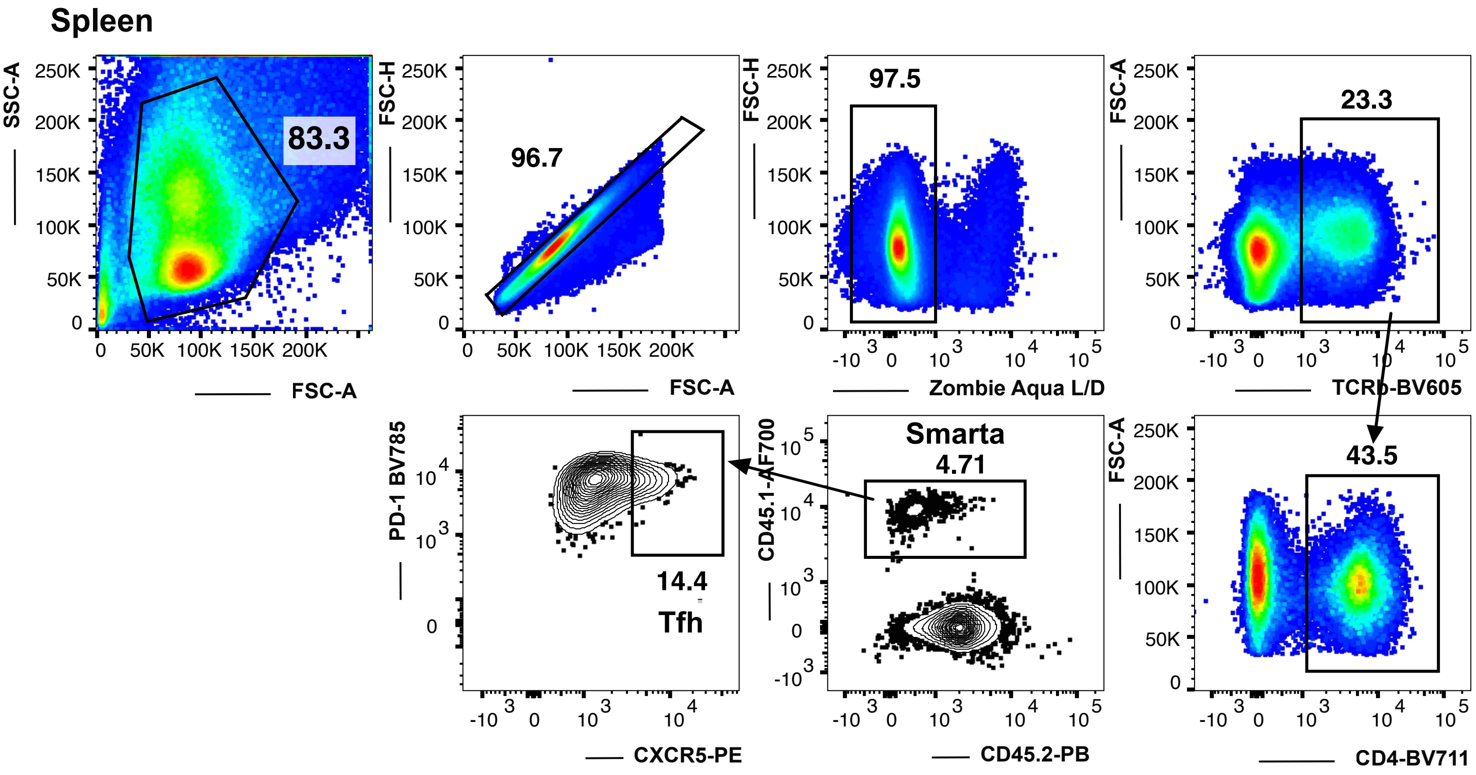

Supplementary Figure 8. Gating strategy for the identification of adoptively transferrred Smarta Tfh cells.

Representative flow cytometry plots showing gating strategy for the identification of adoptively transferred Smarta Tfh cells in the spleen.

Supplementary Table 1

Designation	Sequence (5´ - 3´)	Specificity
Isol46_Exonucl.2_fwd	CGGATCGTAAAGCTCTGTTGTAAG	I46
Isol46_Exonucl.3_rev	GCTACCGTCACTCCCATAGCA	
Probe3_Isol46	FAM-AAGAACGGCTCATAGAGG-BHQ1	
Isol49_Exonucl._fwd	GCACTGGCTCAACTGATTGATG	I49
Isol49_Exonucl._rev	CCGCCACTCACTGGTGATC	
Probe_Isol49	HEX-CTTGCACCTGATTGACGA-BHQ1	
YL58_Exonucl._fwd	GAAGAGCAAGTCTGATG TGAAAGG	YL58
YL58_Exonucl._rev	CGGCACTCTAGAAAAACA GTTTCC	
Probe_YL58	FAM-TAACCCCAGGACTGCAT-BHQ1	
YL27_Exonucl.2_fwd	TCAAGTCAGCGGTAAAAA TTCG	YL27
YL27_Exonucl.2_rev	CCCACTCAAGAACATCAG TTTCAA	
Probe2_YL27	HEX-CAACCCCGTCGTGCC-BHQ1	
YL31_Exonucl.2_fwd	AGGCGGGATTGCAAGTCA	YL31
YL31_Exonucl.3_rev	CCAGCACTCAAGAACTAC AGTTTCA	
Probe2_YL31	FAM-CAACCTCCAGCCTGC-BHQ1	
YL32_Exonucl.2_fwd	AATACCGCATAAGCGCA CAGT	YL32
YL32_Exonucl.2_rev	CCATCTCACACCACCAAA GTTTT	
Probe2_YL32	HEX-CGCATGGCAGTGTGT-BHQ1	
KB1_Exonucl._fwd	CTTCTTTCCTCCCGAGTG CTT	KB1
KB1_Exonucl._rev	CCCCTCTGATGGGTAGG TTACC	
Probe_KB1	FAM-CACTCAATTGGAAAGAGGAG-BHQ1	
YL2_Exonucl._fwd	GGGTGAGTAATGCGTGA CCAA	YL2
YL2_Exonucl._rev	CGGAGCATCCGGTATTA CCA	
Probe_YL2	HEX-CGGAATAGCTCCTGGAAA-BHQ1	
KB18_Exonucl.2_fwd	TGGCAAGTCAGTAGTGA AATCCA	KB18
KB18_Exonucl.2_rev	TCACTCAAGCTCGACAGT TTCAA	
Probe2_KB18	FAM-CTTAACCCATGAACTGC-BHQ1	
YL44_Exonucl._fwd	CGGGATAGCCCTGGGAAA	YL44
YL44_Exonucl._rev	GCGCATTGCTGCTTTAAT CTTT	
Probe_YL44	HEX-TGGGATTAATACCGCATAGTA-BHQ1	
YL45_Exonucl._fwd	AGACGGCCTTCGGGTTGTA	YL45
YL45_Exonucl._rev	CGTCATCGTCTATCGGTA TTATCAA	
Probe_YL45	FAM-ACCACTTTTGTAGAGAACGA-BHQ1	
Isol48_Exonucl._fwd	GGCAGCATGGGAGTTTGCT	I48
Isol48_Exonucl._rev	TTATCGGCAGGTTGGATA CGT	
Probe_Isol48	HEX-CAAACTTCCGATGGCGAC-BHQ1	

Supplementary Table 2

Antibodies	Source	Identifier
Alexa Fluor® 488 anti-mouse CD8a (clone 53-6.7)	Biolegend	Cat# 100723
Alexa Fluor® 488 anti-mouse IFN- γ (clone XMG1.2)	BD Biosciences	Cat# 557724
Alexa Fluor® 700 anti-mouse CD3e (clone 17A2)	Biolegend	Cat# 100216
Alexa Fluor® 700 anti-mouse CD45 (clone 30-F11)	Biolegend	Cat# 103127
Alexa Fluor® 700 anti-mouse CD45.1 (clone A20)	Biolegend	Cat# 110724
Alexa Fluor® 700 anti-mouse CD8a (clone 53-6.7)	eBioscience	Cat# 56-0081-82
APC anti-mouse CD62L (clone MEL-14)	BD Biosciences	Cat# 553152
APC anti-mouse TNF-α (clone MP6-XT22)	eBiosciences	Cat# 17-7321-82
APC-eFluor® 780 anti-mouse CD8a (clone 53 6.7)	eBiosciences	Cat# 47-0081-82
Brilliant Violet 421™ anti-mouse KLRG1 (clone 2F1)	Biolegend	Cat# 138413
Brilliant Violet 605™ anti-mouse CD4 (clone RM4-5)	Biolegend	Cat# 100547
Brilliant Violet 711™ anti-mouse CD4 (clone RM4-5)	Biolegend	Cat# 100549
Brillian Violet 711™ anti-mouse CD8a (clone 53-6.7)	Biolegend	Cat# 100759
Brilliant Violet 785™ anti-mouse CD4 (clone GK1.5)	Biolegend	Cat# 100453
Brilliant Violet 785™ anti-mouse CD44 (clone IM7)	Biolegend	Cat# 103059
Brilliant Violet 785™ anti-mouse CD279 (PD-1) (clone 29F.1A12)	Biolegend	Cat# 135225
eFluor660 anti-mouse Gata3 (clone TWAJ)	eBioscience	Cat# 50-9966-42
FITC anti-mouse CD3e (clone 145-2C11)	BD Biosciences	Cat# 553062
Pacific Blue anti-mouse CD4 (clone GK1.5)	Biolegend	Cat# 100428
Pacific Blue anti-mouse CD45.2 (clone 104)	Biolegend	Cat# 109820
Pacific Blue anti-mouse Helios (clone 22F6)	Biolegend	Cat# 137220
PE anti-mouse CD127 (IL-7Rα) (clone A7R34)	Biolegend	Cat# 135009
PE anti-mouse Granzyme B (clone QA16A02)	Biolegend	Cat# 372208
PE anti-mouse RORgt (clone AFKJS-9)	Biolegend	Cat# 12-6988-82
PE/Cyanine 7 anti-mouse CD4 (clone GK1.5)	Biolegend	Cat# 100421
PE/Cyanine 7 anti-mouse CD279 (PD-1) (clone J43)	eBioscience	Cat# 25-9985-80
PE/Cyanine 7 anti-T-bet (clone 4B10)	Biolegend	Cat# 644824
PerCP-Cyanine 5.5 anti-mouse CD4 (clone RM4-5)	Biolegend	Cat # 550954
PerCP-Cyanine 5.5 anti-mouse CD45.2 (clone 104)	eBioscience	Cat# 45-0454-82
PerCP-Cyanine 5.5 anti-mouse Foxp3 (clone FJ-16s)	eBioscience	Cat# 45-5773-82
Purified Rat Anti-Mouse CD16/CD32 (Mouse BD Fc Block™) (clone 2.4G2)	BD Pharmingen™	Cat# 553142
InVivo MAb anti-mouse CD4 (clone GK1.5)	BioXCell	Cat# BE0003-1
InVivo MAb rat IgG2b isotype control, anti-keyhole limpet hemocyanin	BioXCell	Cat# BE0090
Super Bright® 600 anti-mouse TCR beta (clone H57-597)	eBioscience	Cat# 63-5961-82
Zombie Aqua Fixable Viability Kit	Biolegend	Cat# 423101
