# Supplementary material for: A specific microbial consortium enhances Th1 immunity, improves LCMV viral clearance but aggravates LCMV disease pathology in mice
Source: Nat Commun. 2025 Apr 25;16:3902. doi: 10.1038/s41467-025-59073-x (PMC12022176; doi:10.1038/s41467-025-59073-x)
Supplement: Supplementary file 2 — Reporting Summary [file 41467_2025_59073_MOESM2_ESM.pdf]

nature portfolio

Corresponding author(s): Dietmar Zehn, Caspar Ohnmacht

Last updated by author(s): 12 of August 2024

Reporting Summary

Nature Portfolio wishes to improve the reproducibility of the work that we publish. This form provides structure for consistency and transparency in reporting. For further information on Nature Portfolio policies, see our Editorial Policies and the Editorial Policy Checklist.

Statistics

For all statistical analyses, confirm that the following items are present in the figure legend, table legend, main text, or Methods section.

n/a	Confirmed	
☐	☒	The exact sample size (*n*) for each experimental group/condition, given as a discrete number and unit of measurement
☐	☒	A statement on whether measurements were taken from distinct samples or whether the same sample was measured repeatedly
☐	☒	The statistical test(s) used AND whether they are one- or two-sided<br>*Only common tests should be described solely by name; describe more complex techniques in the Methods section.*
☒	☐	A description of all covariates tested
☐	☒	A description of any assumptions or corrections, such as tests of normality and adjustment for multiple comparisons
☐	☒	A full description of the statistical parameters including central tendency (e.g. means) or other basic estimates (e.g. regression coefficient) AND variation (e.g. standard deviation) or associated estimates of uncertainty (e.g. confidence intervals)
☐	☒	For null hypothesis testing, the test statistic (e.g. *F*, *t*, *r*) with confidence intervals, effect sizes, degrees of freedom and *P* value noted<br>*Give P values as exact values whenever suitable.*
☒	☐	For Bayesian analysis, information on the choice of priors and Markov chain Monte Carlo settings
☒	☐	For hierarchical and complex designs, identification of the appropriate level for tests and full reporting of outcomes
☒	☐	Estimates of effect sizes (e.g. Cohen's *d*, Pearson's *r*), indicating how they were calculated

Our web collection on statistics for biologists contains articles on many of the points above.

Software and code

Policy information about availability of computer code

Data collection	- Flow cytometry data was collected using BD FACSDIVA Software (BD BioSciences, Version 8.0.1)<br>- Histological data were analyses were performed on an Olympus microscope (BX53, field number 22), scanned with an Aperio AT2 scanner and representative images were taken using ImageScope v. 12.4.6.5003.<br>- Imaging data was collected using AxioScan 7 (Zeiss)<br>- 16S rRNA Sequencing was done by using the R pipeline Rhea and Namco<br>- qPCR of bacteria Data were analyzed with the LightCycler96® software package (Roche).<br>- For the scRNA sequencing cells were measured with a LSR II flow cytometer (BD)
Data analysis	- Read alignment and gene counting for scRNA sequencing analysis were performed with 10x Genomics Cell-Ranger v7.1. 0 88, using default parameters and pre-built mouse reference v2020-A (10x Genomics) based on mm10 GRCm38.p6 (release 98) and annotation from GENCODE Release M23. Downstream analysis was performed in R v4.3.0 with the R package Seurat v4.3.0 89.<br>- FlowJo software (Tree Star, Inc.), Version 10 was used for analysis of flow cytometry data<br>- Microsoft Excel (Version 2403) was used for table creation<br>- Microsoft PowerPoint (Version 2430) was used for experimental setup<br>- GraphPad Prism (Version 10) was used for graphical and statistical analysis

For manuscripts utilizing custom algorithms or software that are central to the research but not yet described in published literature, software must be made available to editors and reviewers. We strongly encourage code deposition in a community repository (e.g. GitHub). See the Nature Portfolio guidelines for submitting code & software for further information.

Data

Policy information about availability of data

All manuscripts must include a data availability statement. This statement should provide the following information, where applicable:

- Accession codes, unique identifiers, or web links for publicly available datasets
- A description of any restrictions on data availability
- For clinical datasets or third party data, please ensure that the statement adheres to our policy

The authors declare that the data supporting the findings of this study are available within the paper and its supplementary files. Raw data are available from the authors upon reasonable request. Datasets related to single cell sequencing experiments that were generated and analyzed for the current study have been deposited and made publicly available in the Gene Expression Omnibus under the GEO accession number GSE252214 (https://www.ncbi.nlm.nih.gov/geo/query/acc.cgi?acc=GSE252214)

Research involving human participants, their data, or biological material

Policy information about studies with human participants or human data. See also policy information about sex, gender (identity/presentation), and sexual orientation and race, ethnicity and racism.

Reporting on sex and gender	n/a
Reporting on race, ethnicity, or other socially relevant groupings	n/a
Population characteristics	n/a
Recruitment	n/a
Ethics oversight	n/a

Note that full information on the approval of the study protocol must also be provided in the manuscript.

Field-specific reporting

Please select the one below that is the best fit for your research. If you are not sure, read the appropriate sections before making your selection.

☒ Life sciences ☐ Behavioural & social sciences ☐ Ecological, evolutionary & environmental sciences

For a reference copy of the document with all sections, see nature.com/documents/nr-reporting-summary-flat.pdf

Life sciences study design

All studies must disclose on these points even when the disclosure is negative.

Sample size	No statistical methods were used to pre-determine the sample size. Most experiments were repeated at least 2 times to ensure an overall sample size of at least 3 per experimental group. The exact n values used to calculate the statistics are provided per experiment presented in the main and supplementary figure legends of the manuscript. For single cell RNA sequencing, cells from three to five mice were pooled for each group in order to obtain sufficient cell numbers and avoid cell-isolation biases. According to the 3R principle, the minimal replicate number sufficient to ascertain statistics by unpaired t-test or one-way ANOVA was chosen.
Data exclusions	Single cell RNA sequencing analysis:<br>Cells with >1,000 genes, <10% mitochondrial genes, and UMI counts within the values of the 2nd and 98th quantiles were retained, along with genes detected in ≥3 cells. Filtered read counts from each sample were normalized independently with sctransform v0.3.5 90 using the glmGamPoi method 91 and vst.flavor v2. Integration features were identified from top 1000 highly variable genes excluding mitochondrial, ribosomal and TCR genes before calculation. Anchors between cells from different samples were identified on the integration features using reciprocal PCA and selected using the first 20 dimensions and two neighbours. Data integration was performed considering 50 neighbours to weight the anchors. PCA was calculated for the integrated data on the top 1000 highly variable genes. Both KNN graph and UMAP (spread 1, min.distance 0.3) were computed on the 30 nearest neighbours and first 20 PCA dimensions. Louvain clusters were identified using the Shared nearest neighbour (SNN) modularity optimization-based algorithm at resolution 0.5. Differential expression was performed using the Wilcoxon Rank Sum test and Bonferroni correction.<br><br>LCMV Cl-13 Infection:<br>Successful LCMV Cl-13 infection of gnotobiotic animals was routinely ascertained by PD-1 staining of CD8+ T cells and mice with low PD-1 expression were exluded from analysis.
Replication	Number of replicates is indicated in each Figure legend. In general, data presented are the result of at least 2 independent experiments with multiple biological replicates. All attempts for replication were successful..

Randomization	Mice were housed in the same room and/or isolators in the respective animal facility. Sex- and age-matched animals were used for experiments whenever possible.
Blinding	The investigators were not blinded during collection of animal tissues due to requirements for cage identification and labeling for treatment purposes. In all experiments, samples were processed simultaneously or in parallel.

Reporting for specific materials, systems and methods

We require information from authors about some types of materials, experimental systems and methods used in many studies. Here, indicate whether each material, system or method listed is relevant to your study. If you are not sure if a list item applies to your research, read the appropriate section before selecting a response.

Materials & experimental systems

n/a	Involved in the study
☐	☒ Antibodies
☒	☐ Eukaryotic cell lines
☒	☐ Palaeontology and archaeology
☐	☒ Animals and other organisms
☒	☐ Clinical data
☒	☐ Dual use research of concern
☒	☐ Plants

Methods

n/a	Involved in the study
☒	☐ ChIP-seq
☐	☒ Flow cytometry
☒	☐ MRI-based neuroimaging

Antibodies

Antibodies used

All antibody details are listed in Material and Methodes and also listed below:
Antigen-Clone-Fluorochrome-Source-Catalog number:
All antibody details are listed in Material and Methodes and also listed below:
Antigen-Clone-Fluorochrome-Source-Catalog number:
Antibodies Source Identifier
Alexa Fluor® 488 anti-mouse CD8a (clone 53-6.7) Biolegend Cat# 100723
Alexa Fluor® 488 anti-mouse IFN- γ (clone XMG1.2) BD Biosciences Cat# 557724
Alexa Fluor® 700 anti-mouse CD3e (clone 17A2) Biolegend Cat# 100216
Alexa Fluor® 700 anti-mouse CD45 (clone 30-F11) Biolegend Cat# 103127
Alexa Fluor® 700 anti-mouse CD45.1 (clone A20) Biolegend Cat# 110724
Alexa Fluor® 700 anti-mouse CD8a (clone 53-6.7) eBioscience Cat# 56-0081-82
APC anti-mouse CD62L (clone MEL-14) BD Biosciences Cat# 553152
APC anti-mouse TNF-α (clone MP6-XT22) eBiosciences Cat# 17-7321-82
APC-eFluorâ 780 anti-mouse CD8a (clone 53 6.7) eBiosciences Cat# 47-0081-82
Brilliant Violet 421™ anti-mouse KLRG1 (clone 2F1) Biolegend Cat# 138413
Brilliant Violet 605™ anti-mouse CD4 (clone RM4-5) Biolegend Cat# 100547
Brilliant Violet 711™ anti-mouse CD4 (clone RM4-5) Biolegend Cat# 100549
Brillian Violet 711™ anti-mouse CD8a (clone 53-6.7) Biolegend Cat# 100759
Brilliant Violet 785™ anti-mouse CD4 (clone GK1.5) Biolegend Cat# 100453
Brilliant Violet 785™ anti-mouse CD44 (clone IM7) Biolegend Cat# 103059
Brilliant Violet 785™ anti-mouse CD279 (PD-1) (clone 29F.1A12) Biolegend Cat# 135225
eFluor660 anti-mouse Gata3 (clone TWAJ) eBioscience Cat# 50-9966-42
FITC anti-mouse CD3e (clone 145-2C11) BD Biosciences Cat# 553062
Pacific Blue anti-mouse CD4 (clone GK1.5) Biolegend Cat# 100428
Pacific Blue anti-mouse CD45.2 (clone 104) Biolegend Cat# 109820
Pacific Blue anti-mouse Helios (clone 22F6) Biolegend Cat# 137220
PE anti-mouse CD127 (IL-7Rα) (clone A7R34) Biolegend Cat# 135009
PE anti-mouse Granzyme B (clone QA16A02) Biolegend Cat# 372208
PE anti-mouse RORgt (clone AFKJS-9) Biolegend Cat# 12-6988-82
PE/Cyanine 7 anti-mouse CD4 (clone GK1.5) Biolegend Cat# 100421
PE/Cyanine 7 anti-mouse CD279 (PD-1) (clone J43) eBioscience Cat# 25-9985-80
PE/Cyanine 7 anti-T-bet (clone 4B10) Biolegend Cat# 644824
PerCP-Cyanine 5.5 anti-mouse CD4 (clone RM4-5) Biolegend Cat # 550954
PerCP-Cyanine 5.5 anti-mouse CD45.2 (clone 104) eBioscience Cat# 45-0454-82
PerCP-Cyanine 5.5 anti-mouse Foxp3 (clone FJ-16s) eBioscience Cat# 45-5773-82
Purified Rat Anti-Mouse CD16/CD32 (Mouse BD Fc Block™) (clone 2.4G2) BD Pharmingen™ Cat# 553142
InVivoMAb anti-mouse CD4 (clone GK1.5) BioXCell Cat# BE0003-1
InVivoMAb rat IgG2b isotype control, anti-keyhole limpet hemocyanin BioXCell Cat# BE0090
Super Brightâ 600 anti-mouse TCR beta (clone H57-597) eBioscience Cat# 63-5961-82
Zombie Aqua Fixable Viability Kit Biolegend Cat# 423101

Validation	Commercially available antibodies have been validated by their respective vendors for species reactivity and application in flow cytometry. Validation data are available on the manufacturer's website using the catalog number of each product.<br>Single color controls and Fluorescence Minus One Controls were used to ensure high quality data.

Animals and other research organisms

Policy information about studies involving animals; ARRIVE guidelines recommended for reporting animal research, and Sex and Gender in Research

Laboratory animals	C57BL/6 mice (CD45.2+) animals were purchased from Charles River (France) and maintained under specific pathogen-free (SPF) conditions at the Technical University of Munich, Germany. SMARTA TCRαβ (CD45.1+) transgenic mice were kindly provided by A. Oxenius and bred and maintained under SPF conditions at the Technical University of Munich, Germany. Germ-free (GF) mice and gnotobiotic mice stably colonized with the Oligo-Mouse-Microbiota 12 (OMM12) mice were bred and maintained at the Central Animal Facility of Hannover Medical School, Germany, according to standard operating procedures. OMM12 mice harbor the following bacterial strains: Acutalibacter muris KB18, Flavonifractor plautii YL31, Enterocloster clostridioforme (former Clostridium clostridioforme) YL32, Blautia coccoides YL58, Clostridium innocuum I46, Limosilactobacillus reuteri (former Lactobacillus reuteri) I49, Enterococcus faecalis KB1, Bacteroides caecimuris I48, Muribaculum intestinale YL27, Bifidobacterium animalis YL2, Turicimonas muris YL45 and Akkermansia muciniphila YL44. Gnotobiotic mice were kept in sterile isolators throughout the course of the experiments and received pelleted 50 kGy gamma-irradiated feed (Ssniff Spezialdiäten, Soest, Germany) and autoclaved water ad libitum. Mice housed in isolators were constantly monitored according to recommendations for maintaining gnotobiotic colonies and FELASA recommendations and were routinely tested for contaminations. Samples were collected under sterile conditions using sterile forceps and placed into sterile tubes to minimize the risk of contamination by environmental bacteria and fungi during sampling. All interventions were carried out with male or female mice at least 6-week-old in compliance with the Technical University of Munich institutional regulations and were approved by the local authority (ROB-55.2-2532.Vet_02-19-137). All mice were exposed to a 12:12 h light-dark cycles with food and water administration ad libitum. Animals were randomly assigned to experimental groups which were non-blinded, and no specific method was used to calculate sample sizes.
Wild animals	No wild animals were used in this study.
Reporting on sex	Mice were age- and sex-matched. Male and female mice were used for all experiments.
Field-collected samples	No field-collected samples were used in this study
Ethics oversight	All animal procedures were performed in accordance with national and institutional guidelines for animal welfare and approved by the Regierung of Oberbayern.

Note that full information on the approval of the study protocol must also be provided in the manuscript.

Plants

Seed stocks	*Report on the source of all seed stocks or other plant material used. If applicable, state the seed stock centre and catalogue number. If plant specimens were collected from the field, describe the collection location, date and sampling procedures.*
Novel plant genotypes	*Describe the methods by which all novel plant genotypes were produced. This includes those generated by transgenic approaches, gene editing, chemical/radiation-based mutagenesis and hybridization. For transgenic lines, describe the transformation method, the number of independent lines analyzed and the generation upon which experiments were performed. For gene-edited lines, describe the editor used, the endogenous sequence targeted for editing, the targeting guide RNA sequence (if applicable) and how the editor was applied.*
Authentication	*Describe any authentication procedures for each seed stock used or novel genotype generated. Describe any experiments used to assess the effect of a mutation and, where applicable, how potential secondary effects (e.g. second site T-DNA insertions, mosiacism, off-target gene editing) were examined.*

Flow Cytometry

Plots

Confirm that:

- [x] The axis labels state the marker and fluorochrome used (e.g. CD4-FITC).
- [x] The axis scales are clearly visible. Include numbers along axes only for bottom left plot of group (a 'group' is an analysis of identical markers).
- [x] All plots are contour plots with outliers or pseudocolor plots.
- [x] A numerical value for number of cells or percentage (with statistics) is provided.

Methodology

Sample preparation	Cell isolation from tissues for FACS analysis<br>Mice were euthanized and small Intestine and spleen were harvested. Splenocytes were isolated by manually mashing spleens through a 100-µm nylon cell strainer (BD Falcon). Red blood cells were lysed with a hypotonic ACK buffer. Cell

suspensions were then washed with RPMI medium (RPMI-1640 supplemented with 10% fetal bovine, 2 mM L-glutamine, 1% penicillin-streptomycin, 1 mM sodium pyruvate, 50 nM beta-mercapthoethanol) and then centrifuged 5 min at 500 x g at 4 ° C. Splenocytes were resuspended and used for flow cytometry staining. For cell isolation of the lamina propria, the first 11 cm of the small intestine (duodenum) were used to isolate lamina propria lymphocytes as previously described 3. Briefly, Peyer's Patches were removed, and the intestine was flushed with cold PBS. The tissue was then cut longitudinally and incubated for 30 min in 30 mM EDTA in PBS at pH 8 on ice. The tissue was then shaken vigorously in a repetitive manner and washed with PBS and the supernatant was removed until the solution appeared clear. Remaining tissue was minced into small pieces, and digested in RPMI containing 25 mM HEPES, 0.05 mg/ml collagenase D (Roche), and 10 µg/ml DNase I (Sigma-Aldrich) at 37 °C for 30 min for two consecutive rounds. Between incubation steps, the tissue was pipetted several times up and down. The supernatant was transferred to a new tube and replaced with new digestion media. The combined supernatant was then filtered through a 70-µm cell strainer and centrifuged at 500 x g for 10 min. The cell pellet was then resuspended in 40% Percoll (GE Healthcare) solution and layered onto an 80% Percoll layer. The Percoll gradient was run at 1500 x g at room temperature for 15 min. The interlayer containing lamina propria mononuclear cells was collected and washed prior to further analysis.

Flow cytometry
Restimulation and intracellular cytokine staining
For intracellular cytokine staining, isolated lymphocytes were resuspended in complete RPMI and cultured in 96-well U-bottom plates (3*106 cells per well) at 37 °C with 5% CO2 for 30 min and stimulated at 2 µg/ml with the LCMV GP61-80 peptide, 10 ng/ml PMA and 1 µM ionomycin (Sigma Aldrich) or kept in complete RPMI as unstimulated control. Then, 7 µg/ml Brefeldin A (Sigma Aldrich) was added, and cells were incubated for another 3 hours. Cells were harvested and washed with PBS. In some cases, samples from two animals of the same group were pooled to reach the appropriate number of cells per well.

Intracellular antibody staining and flow cytometry analysis
Intracellular staining was routinely performed with the Foxp3/transcription factor staining kit (eBioscience) according to the manufacturer's instructions. Before staining, all cell preparations were incubated with 3.3 µg/ml rat anti-mouse CD16/32 (Fc receptor block, BD) for 10 min on ice to block unspecific antibody binding. For extracellular staining, the following antibodies were used: anti-mouse CD45.1 Alexa Fluor 700 (Ly5.1, Southern Biotec), CD45.2 Pacific Blue (Ly5.2, Biolegend), TCR Super Bright 600 (H57-597, eBioscience), CD4 Brilliant Violet 711 (RM4-5, Biolegend), PD-1 Brilliant Violet 785 (29F-1A12, Biolegend). Live/dead staining was routinely done using the Zombie AquaTM fixable viability kit (Biolegend). Cells were incubated with extracellular antibodies 30 min on ice and then washed twice with PBS. Cells were then fixed in 150 µl of the provided Fixation/Permeabilization buffer overnight at 4 °C. Before proceeding with intracellular staining, cells were washed again twice with PBS. For intracellular staining the following antibodies were used: anti-mouse IFN-Alexa Fluor 488 (XMG1.2, BD), Granzyme B-PE (QA16A02, Biolegend), TNF-APC (MP6-XT22, eBioscience), T-bet-PE-Cy7 (4B10, Biolegend). Cells were stained for 1 hour at room temperature in the dark. After washing, samples were measured with a LSR II flow cytometer (BD).

Instrument

LSR Fortessa (BD Biosciences), ARIA III Fusion (BD Biosciences),

Software

Data collection: BD FACSDica Software (BD BioSciences)
Data Analysis: FlowJo software (Tree Star, Inc.)

Cell population abundance

Abundance post sort purity was typically >95%, purity was confirmed by post sort flow cytometric re-analysis.

Gating strategy

For analysis of T helper cell populations in SI-LP, cells were defined by gating on SSC-A vs FSC-A plot, doublet exclution was performed using FSC-H vs FSC-A and SSC-H vs SSC-A. Living T helper cells were defined by exclution of Zombie Aqua L/D positive cells, CD45-AF700 positiv, CD3e-FITC positiv, CD4-BV711 positiv and CD8a-APCa780 negativ, Foxp3-PerCPCy5.5 negativ - Th2 cells were defined by RORgt-PE negativ and GATA3-eF660 positiv, Th17 cells were defined as RORgt-PE positiv and GATA3-eF660 negativ. Tregs were defined as Foxp3-PerCPCy5.5 positiv and pTregs as RORgt-PE positiv and Helios-PB negativ.

Single cell suspensions from spleen and SI-LP were analysed for their Th1 population and cytokine secretion by gating on SSC-A vs FSC-A plot, doublet exclution was performed using FSC-H vs FSC-A and SSC-H vs SSC-A. Living T helper cells by exclution of Zombie Aqua L/D positive cells, CD45-AF700 positiv, CD3e-FITC positiv, CD4-BV711 positiv and CD8a-APCa780 negativ, Foxp3-PerCPCy5.5 negativ, Th1-PECy7 high. Cytokine secretion of CD4 T cells was defined by the same gating strategy; IFNg+ and TNFa+ or double positive T helper cells were defined by respective gates/quadrants.

Single cell suspensions from spleen and SI-LP were analysed for SMARTA cells were defined by by gating on SSC-A vs FSC-A plot, doublet exclution was performed using FSC-H vs FSC-A and SSC-H vs SSC-A. Living T helper cells by exclution of Zombie Aqua L/D positive cells, CD3e-FITC positiv, CD4-BV758 positiv and CD45.2-PB negativ/CD45.1 AF700 positiv. Cytokine secretion of CD4 T cells was defined by the same gating strategy; IFNg+ and TNFa+ or double positive T helper cells were defined by respective gates

For the single cell sequencing experiment, single-cell suspension of splenocytes were enriched for CD4+ T cells using a mouse CD4+ T cell enrichment kit according to the manufacturer's instructions. Enriched CD4+ T cells and SI-LP lymphocytes were stained with anti-mouse CD45.1-Alexa Fluor 700, CD4-Brilliant Violet 711 and the non-fixable Live/Dead stain 7-Aminoactinomycin D. Live CD45.1+ virus-specific SMARTA T lymphocytes per group were sorted on a FACS Aria II into tubes containing PBS with 10 % FCS.

Single colour controls and flourescence minus one were used to ensure proper compensation and gating strategies.

☒ Tick this box to confirm that a figure exemplifying the gating strategy is provided in the Supplementary Information.
